# Mobility of oxathiapiprolin in and between tomato plants

**DOI:** 10.1002/ps.7280

**Published:** 2022-11-22

**Authors:** Yigal Cohen, Michal Weitman

**Affiliations:** ^1^ Faculty of Life Sciences Bar Ilan University Ramat Gan Israel; ^2^ Department of Chemistry Bar Ilan University Ramat Gan Israel

**Keywords:** late blight, plant‐to‐plant communication, root exudates, root secretion, systemic translocation, Zorvec, Orondis

## Abstract

**BACKGROUND:**

Oxathiapiprolin (OXPT; FRAC code 49) is a new piperidinyl‐thiazole isooxazoline anti‐oomycete fungicide that targets oxysterol‐binding proteins. The fungicide is known to translocate acropetally from root to shoot to protect plants against fungal attack.

**RESULTS:**

OXPT is ambimobile. It can also translocate basipetally from shoot to root. OXPT exhibits an unprecedented capacity for trans‐plant protection. When two tomato plants are grown in one pot, and one is treated with OXPT (on the stem, leaves or apex), while the other plant and soil surface are adequately covered, both plants become protected against late blight caused by *Phytophthora infestans.*

**CONCLUSION:**

Trans‐plant systemic protection induced by OXPT involves translocation of the fungicide from the shoot of the treated plant to its root, exudation into the soil and uptake by the root of the neighboring untreated plant to protect it against the disease. Liquid chromatography–tandem mass spectrometry analyses confirmed the occurrence of OXPT in root exudates of OXPT‐treated tomato plants in quantities sufficient to protect detached tomato leaves and intact plants against *P. infestans*. © 2022 The Authors. *Pest Management Science* published by John Wiley & Sons Ltd on behalf of Society of Chemical Industry.

## Graphical abstract

Oxathiapiprolin is ambimobile in tomato plants. It moves systemically from the apex or stem to the root of a treated plant, exudes into the soil, is taken up by the root of an adjacent untreated plant and protects it against *Phytophthora infestans*.

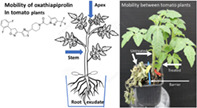

## 1 INTRODUCTION

Oxathiapiprolin (OXPT, piperidinyl‐thiazole isooxazoline, FRAC code 49) is a site‐specific fungicide that targets the oxysterol‐binding protein homolog in oomycetes.^1^ Oxysterol‐binding proteins are implicated in the movement of lipids between the plasma membrane and endoplasmic reticulum, maintaining cell membranes, and the formation of lipids essential for cell survival^2^, ^3^ (FRAC 2022). OXPT is systemic and can protect plants as they grow.^1^ It provides high disease control when applied as a seed treatment (sunflower, tomato, cucumber, and soybean), foliar spray (cucumber, tomato, basil, potato, grapes), or soil drench (tomato, cucumber, pepper, tobacco, basil, potato, soybean, avocado, and citrus). Evidence from studies with tomato,^4^, ^5^ cucumber,^6^, ^7^, ^8^ sunflower,^8^, ^9^, ^10^ basil,^11^ potato,^11^ pepper,^12^ tobacco,^13^ avocado,^14^ citrus,^15^, ^16^ and grapevines^1^ confirms that OXPT is a systemic fungicide that can translocate acropetally in the plant, presumably via the xylem vessels.

In tomato, OXPT applied to the bottom leaves provided high protection against *Phytophthora infestans* in newly developing leaves at 17 days.^17^ When applied to the soil in which young tomato plants were growing, or to seeds, full protection against *P. infestans* was noticed.^17^ In cucumber, OXPT applied to the hypocotyl or soil provided excellent protection against downy mildew caused by *Pseudoperonospora cubensis*.^4^, ^6^ A single application of OXPT (1 mg per plant) to the root of nursery plants grown in multicell trays provided durable systemic protection for up to 4 weeks in tomato against late blight, cucumber against downy mildew, and basil against downy mildew.^5^ OXPT applied to the soil in the field at a dose of 2.5 mg per plant protected potato plants against late blight throughout the season.^11^ OXPT persisted in the treated soil for at least 139 days, providing systemic protection against late blight to subsequent potato crops grown in treated soils.^11^

The objective of this study was to use bioassays and liquid chromatography–tandem mass spectrometry (LC–MS/MS) to explore the venues through which OXPT can translocate in and between tomato plants.

## 2 MATERIALS AND METHODS

### 2.1 Plants

Tomato seeds of *cv* Roter Gnom (RG), deterministic growth type and *cv* Baby, non‐deterministic growth type, were a gift from Syngenta. Plants were grown from seeds in the greenhouse, in multicell trays (cell size 5 × 5 cm, Hishtil) filled with peat/perlite (10:1, v/v) substrate (herein “soil”). Two weeks after sowing, plants were transplanted into 150‐ml pots filled with the same soil, at a density of one or two plants per pot. Unless stated otherwise, plants were used in experiments at approximately 4 weeks after transplanting when they had developed eight true leaves. Each 150‐ml pot contained 45 ± 2.3 g dry soil with water‐holding capacity of 70 ± 3.2 ml.

### 2.2 Fungicide

Formulated OXPT (100 g L^−1^ oil dispersion, Zorvec Enicade™ 100OD) was a gift from DuPont. Pure OXPT (97%) was a gift from Syngenta. The formulated fungicide OXPT was dissolved in water and diluted to a series of tenfold concentration solutions of 0.001 to 1 mg active ingredient (ai) per ml. All concentrations are given as units of ai. The fungicide was applied, in different experiments, to the soil, leaves, stem, or apex (two youngest emerging leaves) of plants grown in 150‐ml pots. When applied to plant organs, adequate care was taken to avoid any contact between the fungicide and the soil or plant organs other than those being studied.

### 2.3 Pathogen, inoculation, and bioassay

Isolate 164 of *P. infestans* (collected in March 2016 from potato at Nirim, Western Negev, Israel) was used in all experiments. This isolate is resistant to mefenoxam and belongs to genotype 23_A1. The pathogen was propagated on detached tomato leaves in moistened 14‐cm diameter Petri dishes kept in a growth chamber at 20°C (14 h light/day, 100 μEinstein s^−1^ m^−2^). For inoculation of intact plants, fresh sporangia were collected from detached sporulating tomato leaves into ice cold distilled water (DW), adjusted to 5 × 10^3^ sporangia ml^−1^, and sprayed with a glass atomizer onto healthy (treated or untreated) tomato plants. Inoculated plants were kept wet in a dew chamber (18°C, darkness) overnight and then in a growth chamber as above. Disease records were taken at 7 days post inoculation (dpi) (unless stated otherwise) by visual estimation of the percent infected leaf area.

To estimate the inhibitory activity of root exudates against *P. infestans* five detached tomato leaflets were placed, lower surface uppermost, on a moistened filter paper in a 14‐cm Petri dish and sprayed with test exudate using a glass atomizer (approximately 0.5 ml per leaflet). Leaflets sprayed with DW served as controls. Leaflets were then drop‐inoculated with sporangial suspension (5 × 10^3^ sporangia ml^−1^) of isolate 164 of *P. infestans*, using six 20‐μl droplets per leaflet. Plates were incubated overnight in a dew chamber (as above) and then in a growth chamber (as above). The proportion of leaflet area showing infection with *P. infestans* was visually estimated at 7 dpi.

### 2.4 Fungicide application

To study its mobility, OXPT was applied to the soil, leaves, stem, or apex of tomato plants grown singly in 150‐ml pots.

#### 2.4.1 Application to the root

One milliliter of OXPT solution (0.001–1000 μg ml^−1^) was applied to the soil surface around the stem base of potted tomato plants. A 5‐ml aliquot of DW was added to each pot five times at 1 h intervals to facilitate uptake of the fungicide by the root. Plants were inoculated after 2 days and scored for disease development at 7 dpi.

#### 2.4.2 Application to the leaves

The soil surface of potted plants was covered with aluminum foil. The plants were laid horizontally on paper towels in a chemical hood and the foliage was sprayed with 5 ml of solution containing 0.05–50 μg of OXPT using a fine glass atomizer. The appropriate amount of fungicide per plant was poured into an Eppendorf tube before use. Plants were returned to the vertical position after 3 h when the spray droplets had dried, and then inoculated and scored as described above.

#### 2.4.3 Application to the stem

The soil surface of potted plants was covered with aluminum foil. The plants were laid horizontally on paper towels in a chemical hood and the upper half of the stem surface (7–12 cm long, depending on the size of the plants used in the experiment) was coated with 1–200 μg of OXPT using a fine camel hairbrush. Control plants were left untreated. Plants were returned to the vertical position after 3 h and were inoculated and scored as described above.

#### 2.4.4 Application to the apex

The soil surface of potted plants was covered with aluminum foil and the two youngest emerging leaves were coated with 25–200 μg of OXPT using a fine camel hairbrush. Control plants were left untreated.

### 2.5 Translocation from plant to plant

Two 8‐leaf tomato plants (cv RG or Baby) were grown in a 150‐ml pot. One plant in each pot was treated with 200 μg of OXPT and the other plant was left untreated. In control pots, neither plant was treated with fungicide. OXPT was applied in different experiments to either the leaves, stem, or apex (two youngest emerging leaves) of one plant in each pot as described above. At 1–3 days after treatment the plants were spray‐inoculated with sporangia of *P. infestans* as described above and scored for disease development at 7 dpi.

Translocation from plant to plant was also tested in split pots as follows: pots (150 ml, 7 × 7 × 6 cm) filled with soil mixture were split in half by inserting a vertical plastic plate (7 × 6 × 0.2 cm) in the middle of the pot. Two tomato plants were planted in each half of the pot. When plants had reached the eight‐leaf stage, one was treated on its stem with 200 μg of OXPT and all four plants in the pot were inoculated 2 days later with *P. infestans* and scored for disease development at 7 dpi.

#### 2.5.1 Translocation to drain water

Eight‐leaf tomato plants grown in 150‐ml pots were brought to full water‐holding capacity and then treated on the stem with 200 μg of OXPT. Pots were each placed in a 300‐ml plastic cup and incubated in a growth chamber as above. At 1 day after treatment, a 60‐ml aliquot of DW was gradually added to the soil surface of each pot using a squeeze bottle (without touching the plant). Drain water (about 20 ml per plant) that accumulated in the bottom of the cup was collected and used in bioassays and chemical analysis. This process was repeated at 2 days after treatment.

#### 2.5.2 Translocation from plant to plant in bare‐root plants

Translocation from plant to plant was studied in bare‐root tomato plants as follows: eight‐leaf plants were uprooted from the soil and their root rinsed thoroughly with tap water to remove soil particles. The root of each plant was wrapped in a plastic bag and plants were laid horizontally on paper towels inside a ventilated hood. The plants were treated with OXPT on the leaves, stem, or apex as described above. Control plants were left untreated. At 3 h after treatment, the plastic bags were removed, and the root system of two plants, one treated and the other untreated, were placed in a 300‐ml plastic cup containing 100 ml of DW. Controls consisted of two untreated plants in a cup. Plants were incubated in a growth chamber at 20°C until inoculated with *P. infestans* (and scored for disease development at 7 dpi).

### 2.6 Root exudates of bare‐root plants

The above procedure was repeated except that a single plant (treated or untreated) was placed in each cup containing 50 ml of DW or in a 50‐ml tube containing 25 ml of DW. At various times after treatment, the remaining water (root exudate) was collected (about 25 ml in a cup, 12 ml in a test tube) and replaced with fresh DW. Each root exudate was filtered through a Whatman No. 1 filter paper, then through a 0.45 μm Millipore membrane and used in bioassays and LC–MS/MS analysis. This procedure was modified as required by specific experiments (see Results).

### 2.7 LC–MS/MS analysis

LC–MS/MS was used to identify and quantitate OXPT in stem and root exudates of tomato plants. Calibration curves were obtained by using pure OXPT provided by Syngenta. The compound was identified by its retention time (RT) on the column, the mass of the precursor ion, and the ionic fragments obtained upon breakdown of the molecule in a collision cell.

All analyses were performed with an Agilent Technologies mass spectrometer 6545 QTOF. The machine was equipped with an electrospray ionization interface coupled to a 1260 uniform high‐pressure liquid chromatography (UHPLC) device, a G4204A quaternary pump, G4226A ALS auto‐sampler, and G1316C thermostat‐controlled column compartment. UHPLC was carried out on a ZORBAX RRHD Eclipse Plus C18, 95 Å, 2.1 × 50 mm, 1.8 μm column, with water (0.1% formic acid)/MeCN gradient elution, from 5% to 95% acetonitrile (MeCN) for 10 min at a flow rate of 0.5 ml min^−1^. Ten microliters of each sample or standard was injected into the LC–MS/MS instrument in triplicate and an average peak area of three analyses was calculated. MeCN/water solution was injected as a blank within a sequence of samples to reduce cumulative carryover. Adding formic acid to the needle wash solution reduces carryover. OXPT was monitored by the ion transition 540.149 *m*/*z* [MH]^+^ → 500.134 *m*/*z*. Mass spectral parameters were optimized by varying the fragmentor voltage of the ion source to the scan mode and the collision energy to the product ion mode (MS/MS). Specific parameters of the ion source were readjusted. The electrospray ionization interface was operated in positive mode. The source temperature was set to 300°C and the ion spray voltage was 3.5 kV. High‐resolution accurate mass data were acquired in the scan mode and exported into Mass Hunter Quantitative software for quantitation and identification using qualifier fragment ions.

### 2.8 Data analysis

All experiments were conducted in environmentally controlled growth chambers in a completely randomized design. Experiments were repeated three times or more, with between 3 and 20 replicate plants per treatment in the different experiments. One‐way analysis of variance and Student's *t*‐test were performed to detect significant differences between treatments. The significance of the differences was evaluated by the Fisher test, LSD_05_, and standard deviations from the mean. Statistical analyses of the data were carried out in XLSTAT software. Effective Dose 50 (ED_50_) and Effective Dose 90 (ED_90_) values were derived from log‐probit regression curves using SPSS software.

## 3 RESULTS

### 3.1 Mobility from root, stem, or apex to foliage

**FIGURE 1:**
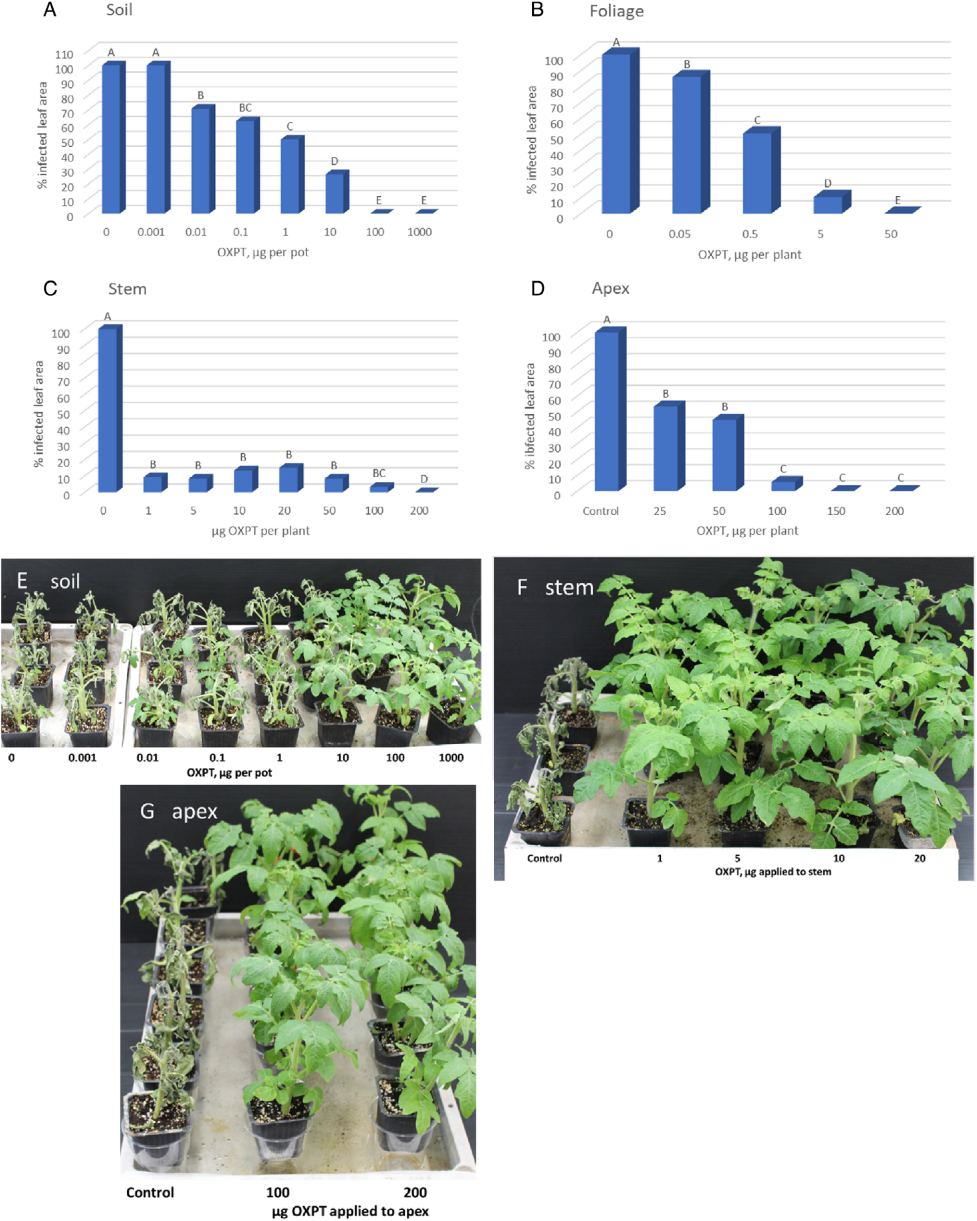
Suppression of late blight development in potted tomato plants by oxathiapiprolin. The fungicide was applied to the soil (A,E), to the foliage (B), to the stem (C,F) and to the apex (D,G). Different letters above columns indicate significant differences between means (*t*‐test, α = 0.05). Plants were photographed at 7 days post inoculation with *Phytophthora infestans*.

Experiments were performed to quantitate the systemic efficacy of OXPT against *P. infestans* when applied to the root, foliage, stem, or apex. One milliliter of solution containing 0.001–1000 μg of OXPT was applied to the soil surface of a 150‐ml pot in which two tomato plants (RG, six‐leaf stage, *n* = 3) were grown. Plants were inoculated 1 day after treatment. The results in Figure 1(A) show that 1, 10, and 100 μg of OXPT per pot provided a mean of approximately 50%, 75% and 100% protection against late blight, respectively, thus confirming previous findings.^17^

When sprayed onto the foliage, 0.5, 5, and 50 μg of OXPT per plant was required to reduce infection by a mean of approximately 50%, 90% and 100%, respectively (Figure 1B).

When the stem of eight‐leaf plants was coated with 1–50 μg of OXPT (plants were inoculated after 2 days), percent protection against late blight at 7 dpi ranged between 85% and 92% (Figure 1C). Application of 100 or 200 μg of OXPT to each stem provided 97% and 100% protection, respectively (Figure 1C).

When the apex of eight‐leaf plants was coated with 25, 50, 100, 150, and 200 μg of OXPT per plant and plants were inoculated for 2 days, percent protection against late blight at 7 dpi was 47%, 55%, 94%, 100% and 100%, respectively (Figure 1D).

The appearance at 7 dpi of the plants treated via the soil, stem or apex is shown in Figure 1(E), (F) and (G), respectively.

The data suggest that OXPT can translocate from the root to the shoot and from the apex to the rest of the foliage. Stem application was the most effective; 1 μg of OXPT was sufficient to provide foliage with 91% protection against the disease.

### 3.2 Mobility from a treated plant to an untreated neighboring plant

We hypothesized that OXPT may translocate from one plant in a pot to its neighboring plant grown in the same pot. To verify this, two plants were grown in a pot and fungicide was applied to the foliage, stem, or apex of one plant only, while adequate care was taken to avoid any contact between the neighboring plant or soil and the fungicide. Drain water was collected from the pots for use in bioassays and chemical analysis, and both plants were inoculated with *P. infestans*.

#### 3.2.1 Foliage spray

**FIGURE 2:**
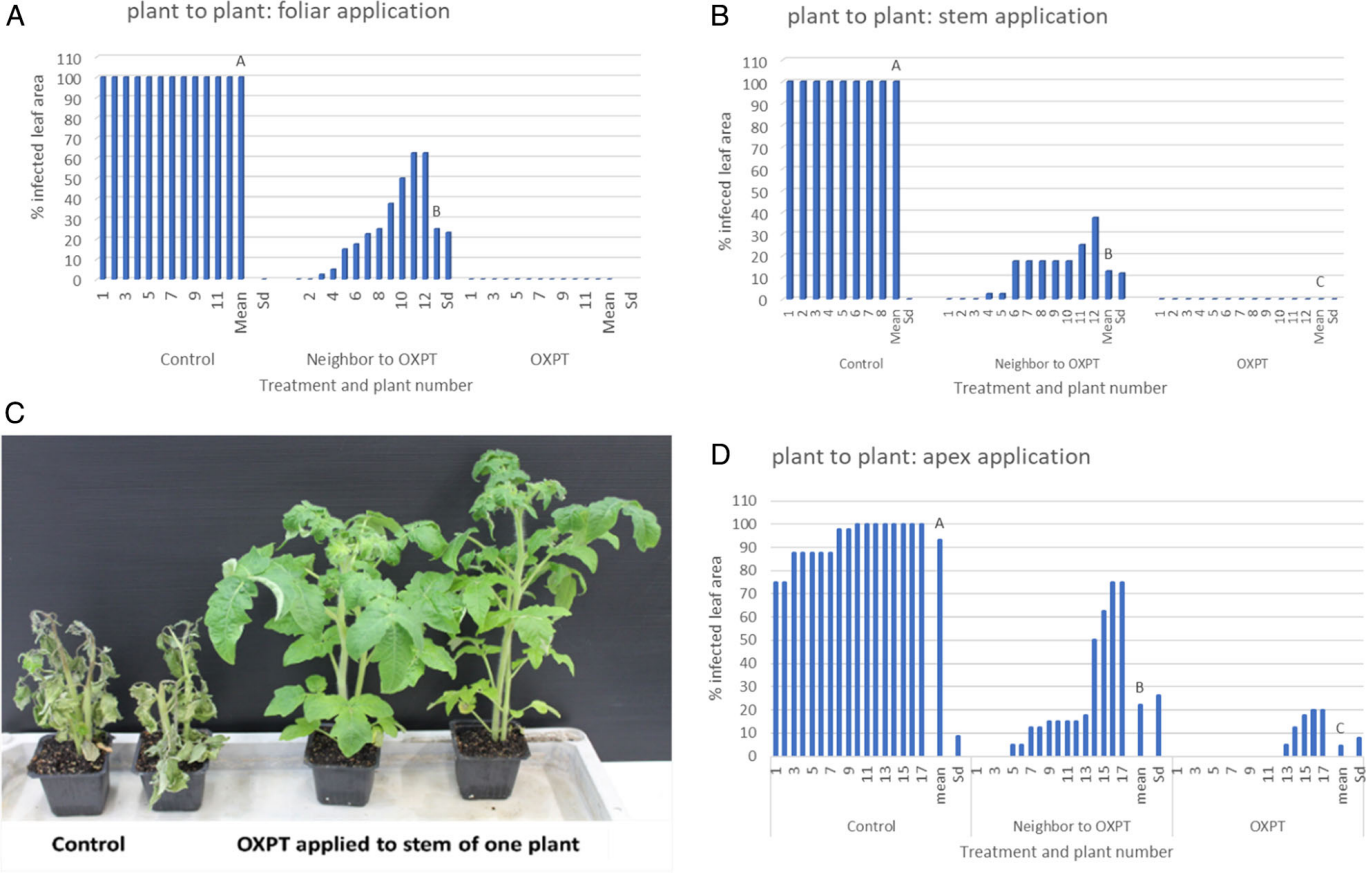
Translocation of oxathiapiprolin (OXPT) from a treated plant to its neighboring untreated plant as evident by the inhibition of late blight development in the untreated neighboring plants. OXPT (200 μg per plant) was applied to the foliage (A), stem (B,C), and apex (D). Different letters above columns indicate significant differences between means (*t*‐test, α = 0.05).

One of the two tomato plants grown in a 150‐ml pot (eight‐leaf, Baby, *n* = 10) was sprayed with 200 μg of OXPT. Plants were inoculated after 3 days and scored at 7 dpi. Control untreated plants were fully devastated by late blight, whereas treated plants showed zero infection (not shown). The disease scores of the individual plants are shown in Figure 2(A) including the mean and standard deviation of the mean. OXPT applied to one plant in a pot provided a mean of 72% protection against blight to the neighboring untreated plant in the same pot (Figure 2A), suggesting translocation of OXPT from plant to plant via the root system.

Translocation from plant to plant was also shown in bare‐root plants. Two bare‐root plants (eight‐leaf RG, *n* = 3), one sprayed with 200 μg of OXPT and the other left untreated, were placed in a 300‐ml plastic cup containing 50 ml of DW. After 2 days the plants were inoculated with *P. infestans* and scored for disease development at 7 dpi. All control plants were fully infected with late blight and all fungicide‐treated plants were free of the disease. All untreated plants that shared a cup with a treated plant showed a disease level of 0%–22.5%. Treating a bare‐root plant with OXPT provided a mean of 82% protection to the untreated plant that shared the same cup.

#### 3.2.2 Stem paste

This experiment was conducted with pairs of eight‐leaf plants (RG) grown together in 150‐ml pots (*n* = 12). The upper half of the stem of one plant in each pot was coated with 200 μg of OXPT and the other plant in the pot was left untreated. In control pots, both plants were left untreated. All plants were inoculated at 2 days after treatment. At 7 dpi, all control inoculated plants were fully devastated by late blight, whereas all stem‐treated inoculated plants were free of late blight symptoms (Figure 2B,C). The untreated plants, neighbors of the treated plants, were segregated in their response to the disease into fully protected and moderately protected plants (Figure 2B). The mean protection provided by OXPT to neighboring plants was 88%.

In another experiment, performed with eight‐leaf plants (RG, 8 control pots and 12 treated pots), late blight development was monitored over a 17‐day period. Mean area under the disease progress curve for control, neighboring, and treated plants was 1444 ± 20, 344 ± 324, and 0, respectively, indicating 76% protection of neighboring plants over the course of the experiment.

Split pot experiments (*n* = 5), in which two plants were grown in each of the two compartments of the split pot, showed that OXPT applied to the stem provided 100% protection to the treated plant and 86% protection to its neighbor grown in the same compartment. The two untreated plants grown in the adjacent compartment were fully devastated by late blight.

Trans‐plant protection was also observed in potted potato and cucumber plants. When one of two or three potato shoots grown from one tuber in a 1‐L pot had its stem coated with 200 μg of OXPT the other shoots showed 61% protection against late blight (data not shown). When the hypocotyl or the leaf of a one cucumber plant, of two grown in a 150‐ml pot, was coated with 50 μg of OXPT both plants showed 94%–96% protection against downy mildew (data not shown).

#### 3.2.3 Apex paste

The apex of one plant (eight‐leaf, RG,) in each 150‐ml pot (*n* = 17) was treated with 200 μg of OXPT, whereas the other plant remained untreated. Plants in control pots remained untreated. Plants were inoculated at 2 days and scored for late blight at 7 dpi. The results showed a mean of 96% protection in treated plants and 76% protection in neighboring plants (Figure 2D).

### 3.3 Vapor activity

To ascertain whether the trans‐plant protection could have resulted from the vapor activity of OXPT, we performed the following bioassay. Nine‐centimeter Petri dishes were divided into three compartments and a detached tomato leaflet was placed in each compartments. In each dish, two leaflets were treated with OXPT (5, 10 or 50 μg per leaflet) and one was inoculated with *P. infestans*. At 7 dpi all inoculated leaflets were fully infected. This and the split pot experiments (see above) support the notion that vapor activity is unlikely to play a role in practice.

### 3.4 Quantification in bioassay and LC–MS/MS

Another plausible explanation for the trans‐plant protection phenomenon is that OXPT translocates basipetally from the treated shoot of a plant to its root, exudes into the soil and is taken up acropetally by the root of the neighboring plant. If true, soil extracts (drain water) from pots in which shoot‐treated plants were grown should contain the fungicide. Biological assays and LC–MS/MS analyses were performed to ascertain whether OXPT is present in these extracts.

**FIGURE 3:**
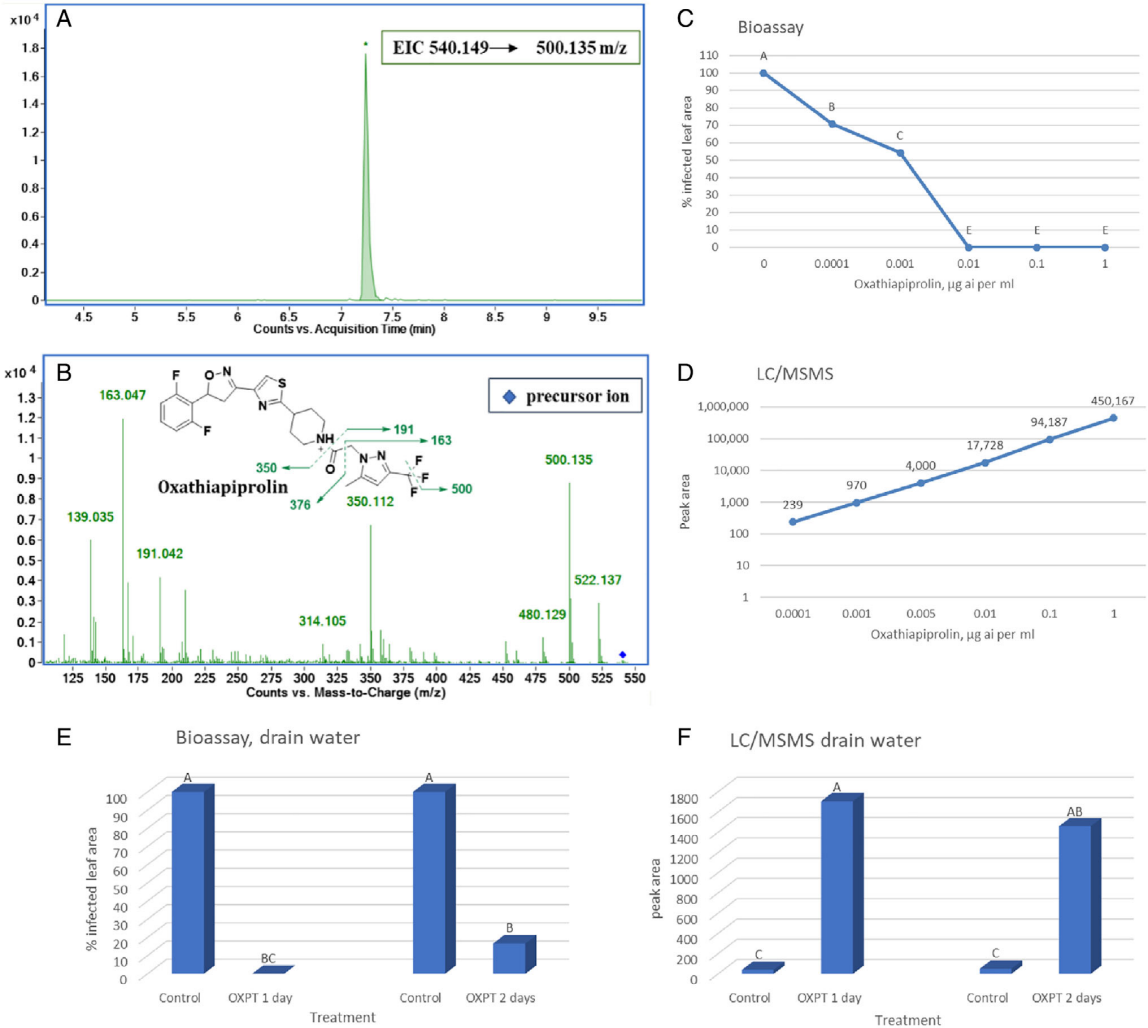
LC–MS/MS of oxathiapiprolin (OXPT). (A) Extracted ion chromatogram (EIC) of the protonated precursor ion [MH]+: OXPT (540.149 → 500.135 *m*/*z*) detected in the water exudates of OXPT‐treated tomato plants. The *x*‐axis represents retention time (min) and the *y*‐axis represents signal intensity. (B) Fragmentation mass spectra by collision‐induced dissociation of OXPT. Precursor ions are shown as a blue diamond. The *x*‐axis represents mass‐to‐charge ratio (*m*/*z*) and the *y*‐axis represents signal intensity. Putative assignments of the characteristic fragment ions of OXPT are shown. (C) Dose‐dependent inhibition of *Phytophthora infestans* by OXPT in detached tomato leaflets. (D) Dose‐dependent peak area of OXPT in liquid chromatography–tandem mass spectroscopy (LC–MS/MS) analysis. (E) Drain waters collected from OXPT‐treated tomato plants inhibit *P. infestans* in bioassays. (F) LC–MS/MS analysis revealed the inhibitory concentration of OXPT in drain waters.

Solutions of 0.0001–1 μg ml^−1^ OXPT (tenfold dilutions) in DW were used for both LC–MS/MS analysis and bioassays. Figure 3(A,B) shows the results of the LC–MS/MS analyses of OXPT. Figure 3(C) shows leaflet infection (*n* = 6) in the bioassay (percent infected leaf area) as scored at 7 dpi. The ED_50_, ED_90_, and minimal inhibitory concentration values were 0.001, 0.013 and 0.01 μg ml^−1^, respectively. LC–MS/MS revealed a straight logarithmic relationship between OXPT concentration and peak area obtained at RT = 7.24 (Figure 3D).

### 3.5 Secretion into drain water

These data were used to estimate the concentration of OXPT in drain water taken from OXPT‐treated tomato plants. OXPT was coated on the stem of intact eight‐leaf RG plants grown singly in 150‐ml pots (*n* = 4). Drain water collected from each pot at 1 and 2 days after treatment caused 100% and 83% inhibition of *P. infestans*, respectively, in the bioassays (Figure 3E). In LC–MS/MS analysis, the drain water produced a peak area of 1706 and 1458 units at RT = 7.24, respectively (Figure 3F), suggesting and OXPT concentration of > 0.001 to < 0.005 μg ml^−1^.

### 3.6 Secretion from roots of bare‐root plants

The above data suggested that OXPT may translocate from plant to plant via the root systems. The following experiments show that this was also true in bare‐root plants whose roots were submerged in DW. Root exudates taken from bare‐root plants that were sprayed with 200 μg of OXPT were inhibitory to *P. infestans* in detached leaf bioassays. LC–MS/MS analysis could detect the inhibitory concentrations of the fungicide in such exudates.

#### 3.6.1 Foliar spray

**FIGURE 4:**
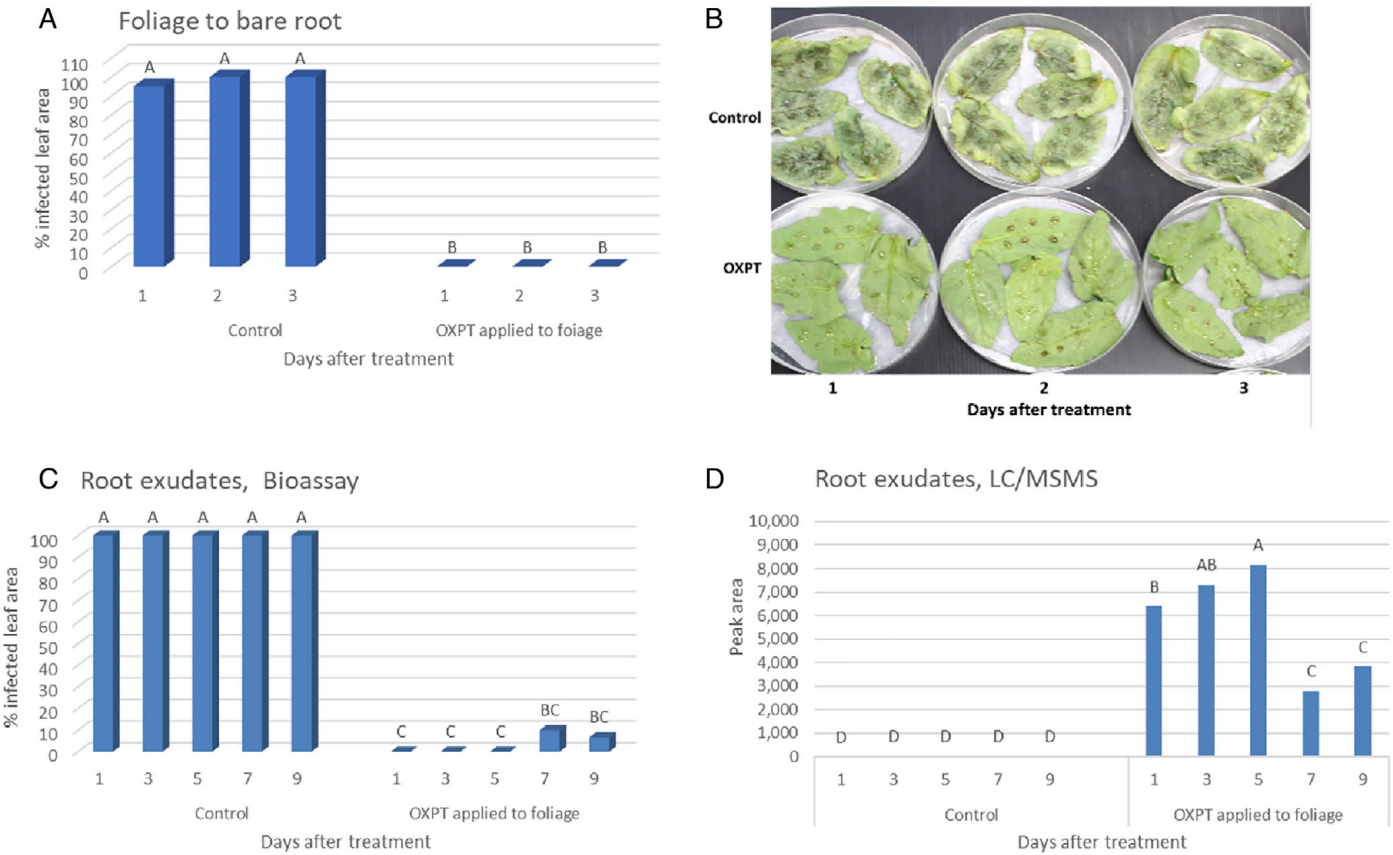
Secretion of oxathiapiprolin (OXPT) from the roots of foliage‐sprayed bare‐root tomato plants. (A,B) Experiment 2: inhibitory effect against *Phytophthora infestans* of root exudates collected at 1, 2, or 3 days after spraying in detached leaf bioassays. (C, D) Experiment 4: bioassay of root exudates collected at 1, 2, and 3 days after spray and their liquid chromatography–tandem mass spectroscopy analyses.

In the first experiment with eight‐leaf bare‐root plants (RG, *n* = 9), root exudates collected at 1, 2, 3, and 4 days after foliar spray were 100%, 100%, 93%, and 77% inhibitory to late blight development, respectively, in detached leaf bioassays. Root exudates collected at these times from untreated control plants allowed 100% infection of the detached leaves. In the second experiment with 12‐leaf bare‐root plants (RG, *n* = 5), root exudates collected at 1, 2, and 3 days after foliar spray were completely inhibitory to late blight development in detached leaf bioassays, whereas root exudates that were collected at these times from untreated plants were fully supportive of disease development (Figure 4A,B). In the third experiment, root exudates taken from treated tomato plants (Baby, ten‐leaf, *n* = 3), were fully inhibitory to *P. infestans* in bioassays. In LC–MS/MS analyses they produced a peak area of 3675 ± 618 units at RT = 7.24 (indicating approximately 0.005 μg ml^−1^ of OXPT in the exudates; Figure 3B). In the fourth experiment, ten‐leaf plants (RG, *n* = 3) were used and root exudates were collected at 1, 3, 5, 7, and 9 days after spraying and used in bioassays and LC–MS/MS analysis. The results showed that root exudates collected from control plants allowed 100% infection with *P. infestans* in detached leaflets bioassays, whereas those collected from OXPT‐treated plants at 1, 3, and 5 days after spraying were fully inhibitory to the disease (Figure 4C). LC–MS/MS analyses revealed the presence of OXPT in the exudates of the treated plants, but not control plants (Figure 4D). Peak areas at 1, 3, and 5 days were significantly higher (approximately 6400–8200 units, > 0.005 to < 0.01 μg ml^−1^) than at 7 and 9 days (approximately 2800–3800 units, > 0.001 to < 0.005 μg ml^−1^) suggesting reduced translocation of the fungicide over time from leaves to root, and/or reduced secretion over time from root to the surrounding water.

#### 3.6.2 Stem paste

Preliminary experiments showed that coating the upper half of the stem of bare‐root plants with 200 μg of OXPT resulted in a significantly stronger inhibitory effect (96%–97%) by their root exudates in bioassays compared with coating with 100 μg of OXPT (47%–64%). Subsequent experiments therefore used 200 μg of OXPT.

**FIGURE 5:**
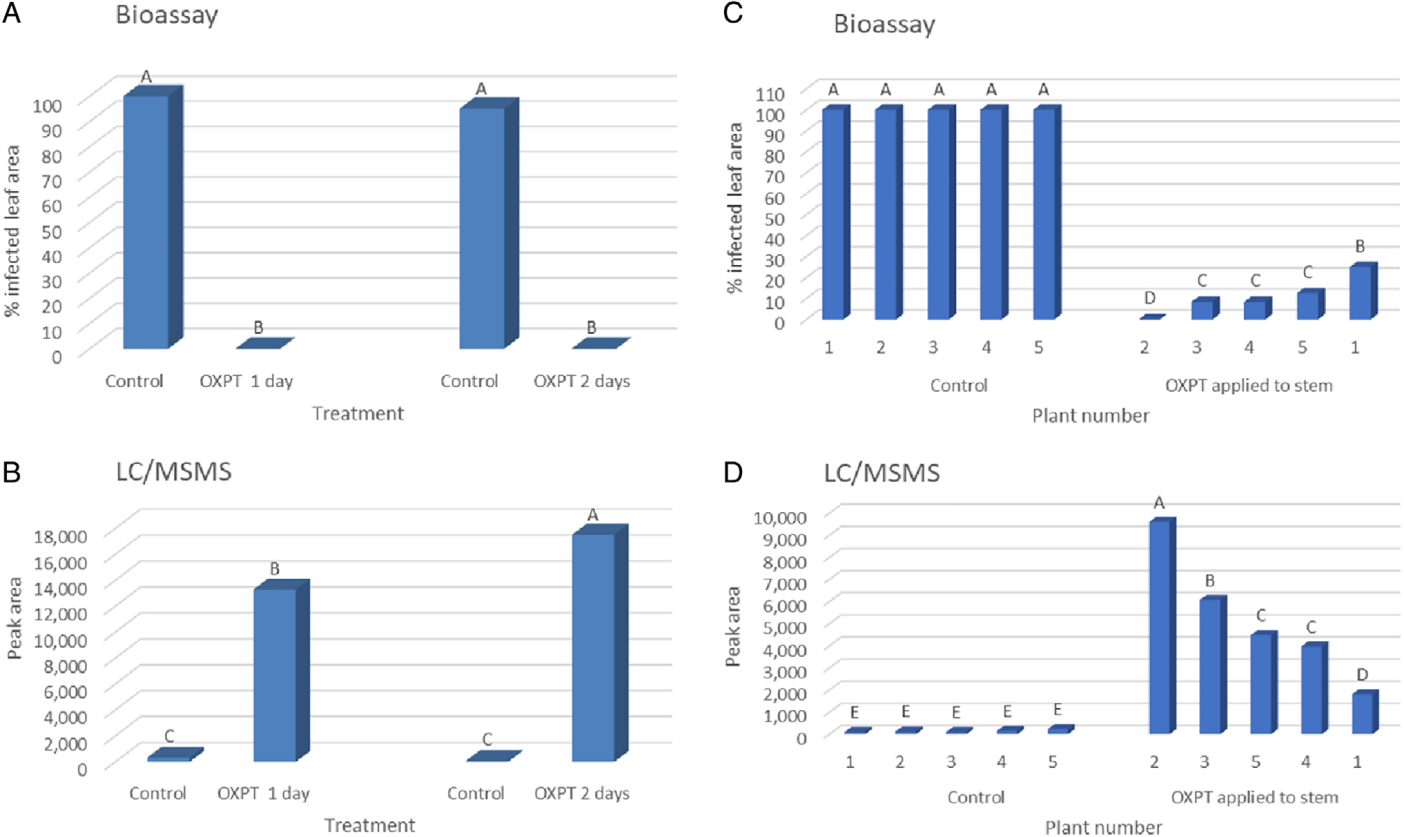
Secretion of oxathiapiprolin (OXPT) from the roots of stem‐treated bare‐root plants. (A) Root exudates collected at 1 or 2 days after treatment suppress late light development in detached leaves bioassays. (B) Liquid chromatography–tandem mass spectroscopy analyses detect OXPT in such root exudates. (C,D) The association between the inhibitory effect of root exudate in bioassays and the quantity of OXPT in the exudates. Different letters above columns indicate significant differences between means (*t*‐test, α = 0.05).

The stems of ten‐leaf bare‐root RG plants (*n* = 3) were coated with 200 μg of OXPT, whereas control plants were left untreated. Root exudates collected at 1 and 2 days after treatment were used for bioassays and LC–MS/MS analysis, and plants were inoculated with *P. infestans* 2 days after treatment. The detached leaf bioassays showed that root exudates taken from plants with OXPT‐treated stems were fully inhibitory, whereas exudates collected from control plants allowed for 95%–100% infection (Figure 5A). LC–MS/MS analyses showed no OXPT in the root exudates of control plants, but confirmed the presence of OXPT (approximately 0.01 μg ml^−1^) in the root exudates of the OXPT‐treated plants (Figure 5B). At 6 dpi, intact control plants were fully devastated by late blight, whereas those coated with the fungicide were fully protected.

The following experiment showed that plants differed in their ability to exude OXPT from their roots. Ten‐leaf bare‐root plants (RG) were treated with 200 μg of OXPT on their stems, and root exudates were collected at 3 days after treatment for bioassay and chemical analysis. The results showed that exudates taken from control plants contained no OXPT and allowed 100% infection with *P. infestans* of the detached leaves in the bioassays, whereas exudates from the roots of OXPT‐treated plants contained various amounts of OXPT ranging from 1800 to 9600 peak area units (> 0.001 to < 0.01 μg) and allowed no or little development of late blight in the bioassays (Figure 5C,D). A correlation coefficient of *R* = −0.9172 was calculated between OXPT content and percent inhibition of *P. infestans* in the bioassay. The data confirmed that OXPT applied to the stem surface of tomato plants can translocate to the foliage to protect it from late blight infection as well as to the root, from which it can exude into the surrounding water.

#### 3.6.3 Apex paste

**FIGURE 6:**
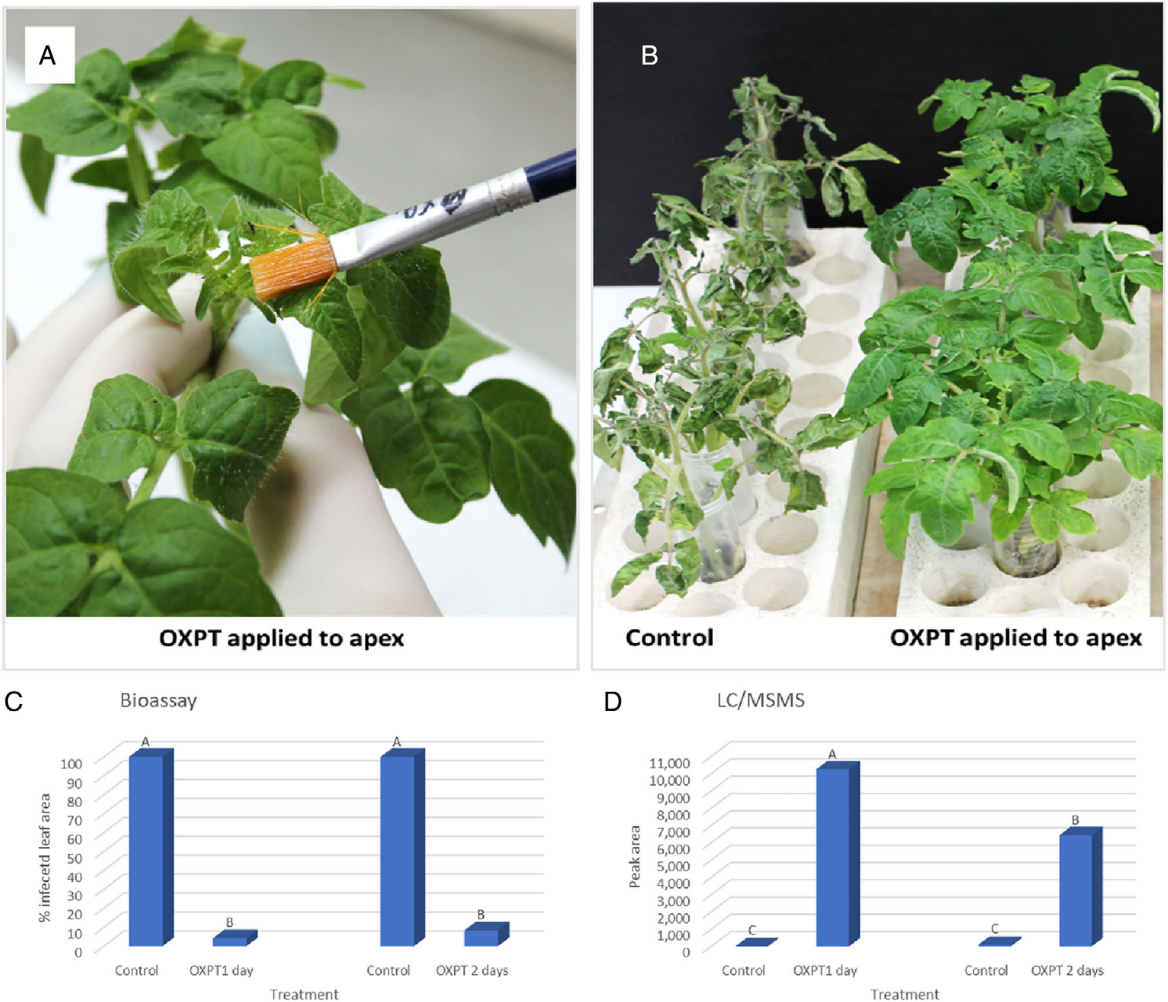
Secretion of oxathiapiprolin (OXPT) from the roots of apex‐treated bare‐root plants. (A) Application of OXPT to the apex. (B) OXPT applied to the apex protected the whole plant against late blight infection. Plants were photographed at 7 days post inoculation with *Phytophthora infestans*. (C) Detached leaf bioassay showing that OXPT exuded from the root of apex‐treated plants caused inhibition of late blight. d‐OXPT is detected in root exudates from apex‐treated plants. Different letters above columns indicate significant differences between means (*t*‐test, α = 0.05).

Bare‐root, eight‐leaf RG tomato plants (*n* = 5) were placed in 50‐ml tubes containing 25 ml of DW and treated on their apex with 200 μg of OXPT using a camel hairbrush (Figure 6A). Control plants were left untreated. At 1 and 2 days after treatment, 10 ml of water was withdrawn and subjected to bioassays and chemical analysis. At 2 days, plants were inoculated with *P. infestans*. The results showed that OXPT applied to the apex provided full protection to the foliage against late blight, whereas control plants were devastated by the blight (Figure 6B). The bioassays showed that exudates collected at 1 and 2 days from roots of the OXPT apex‐treated plants provided 96% and 92% protection, respectively, against *P. infestans* (Figure 6C). LC–MS/MS analyses revealed that the root exudates collected at 1 and 2 days from OXPT apex‐treated plants exhibited peak areas of 10 200 and 6400 units (>0.005 and < 0.01 μg), respectively (Figure 6D), whereas the root exudates of control plants contained no OXPT. The results confirm that OXPT can translocate basipetally from the apex to the foliage and root, from where it can exude into the surrounding water.

## 4 DISCUSSION

OXPT is a newly developed anti‐oomycete systemic fungicide. It can translocate systemically from the root to foliage^8^, ^17^ and from the youngest leaves to newly developing leaves.^1^ The current study confirms that OXPT is ambimobile, also moving basipetally from the apical youngest leaves or stem to the bottom leaves and root. When applied to the shoot (leaves, stem, or apex), OXPT was detected (by bioassay and chemical analysis) in drain water released from the soil of potted tomato plants, suggesting exudation from the root.

This is the reason for the surprising trans‐plant protection described in this study. OXPT applied to the shoot of one plant could protect its neighboring plant (grown in the same pot) from the disease. The degree of such trans‐plant protection was stronger when the fungicide was applied to the stem compared with the leaves or apex. Protection of neighboring plants varied among replicate pots and experiments with an overall mean efficacy of approximately 75%. No such trans‐plant protection was evident when plants were grown in separate adjacent pots, or in split pots.

Vapor phase activity of, for example, mefenoxam was responsible for controlling downy mildew in grapevines fruit clusters.^18^ Vapor activity of OXPT is probably not responsible for the observed trans‐plant protection. The vapor pressure of purified OXPT is low, 1.141 × 10^−6^ Pa at 20°C. Its Henry's Law constant at 20°C, 3.521 × 10^−3^ Pa m^3^ mol^−1^ (calculated using solubility and vapor pressure at 20°C) is low (DuPont data, Jean‐Luc Genet, personal communication). Vapor phase activity was not observed by the manufacturer (Jean‐Luc Genet, personal communication) or by us.

Trans‐plant protection seems, therefore, to result from four consecutive processes: basipetal translocation of OXPT to the root, exudation from the root, uptake by the neighboring root, and acropetal translocation by the neighboring untreated plant. Bioassays and LC–MS/MS analyses conducted with root exudates from treated tomato plants proved the presence of OXPT in the root exudates of treated bare‐root plants.

Bioassays and LC–MS/MS analyses confirmed the presence of OXPT in drain water collected from potted plants in which stem‐ or apex‐treated plants were grown, suggesting basipetal translocation of OXPT from stem to root and secretion from root to soil and out to drain water. OXPT applied directly to the soil of potted tomato plants was effective in suppressing late blight development.^17^

The data show that 10 μg of OXPT applied directly to the soil provided a pair of potted plants with approximately 75% protection against late blight. Such a level of protection was obtained in our plant‐to‐plant mobility experiments, suggesting that a treated plant may secrete approximately 10 μg of OXPT into the soil. Because the amount of OXPT that we applied to a stem or apex is approximately 200 μg, the amount secreted may reach 5% of the applied fungicide. The experiments reported here were conducted using an artificial soil (peat + perlite) yet it is known that OXPT has very little mobility in soil because of its strong adsorption to soil particles (clay and organic matter) leading to little to no leaching. This attribute also explains why high soil application rates are needed for effective disease control (relative to foliar application rates).

The recommended spray rate of formulated OXPT in tomato is 50 g ha^−1^ giving approximately 1000 μg ai per plant. A portion of this may translocate from the sprayed foliage (leaves, apex, stem) to the root and thus confer protection on plants that suffered from poor spray coverage.

The mechanisms that facilitate OXPT exudation from tomato roots are unknown. Passive diffusion and ABC‐transporters may be involved. No other fungicide has been reported to exude from roots of plants treated on the foliage.

Secretion of OXPT from tomato roots therefore seems unique. Most phloem‐mobile pesticides are herbicides. They are either acidic molecules or esters that can be hydrolyzed to an acid once they are in the plant tissues. Several herbicides (glyphosate, thifensulfuron‐methyl, diclofop‐methyl and picloram) exude from the roots of weeds or crop plants.^19^ Some weeds secrete herbicide molecules from roots into the rhizosphere upon being treated and thus develop resistance to that herbicide.^19^ Phloem mobility of a crop protectant is an attribute that contributes positively to its efficacy. Herbicides, insecticides, and fungicides applied foliarly must move to remote plant parts (such as meristems, emerging leaves, roots, and fruits) via the phloem to achieve useful activity.^20^

Phloem‐mobile fungicides are rare. Fosetyl‐Al is the first commercially produced fungicide that possesses substantial capabilities for movement in a basipetal direction from shoot to root.^21^ Phosphonates are effective against foliar and soil‐borne oomycete diseases. Phosphonic acid is efficiently absorbed through roots and leaves by the phosphate transport system and is truly ambimobile within plants; that is, it can translocate throughout the plant within the xylem and phloem.^22^

The fungicide azoxystrobin was reported to translocate in 2 days from a leaflet of tomato to other parts of the plant: 19% to stem, 10% to youngest leaves, 4% to mature leaves and 2% to the root.^23^ When [^14^C]cymoxanil (anti‐oomycete fungicide) was applied to the root of potted tomato plants, 75% and 90% of the label reached the shoot in 1 and 16 h respectively, but when applied to a leaf only 5% of the label reached the other parts of the plant.^24^ The non‐systemic fungicide fludioxonil was recently reported^25^ to control Fusarium T4 in banana by foliar spray of fludioxonil‐loaded glycine methyl ester‐conjugated polysuccinimide nanoparticles (PGA) nanoparticles (FLU@PGA). FLU@PGA sustained the downward delivery of fludioxonil to banana rhizomes and roots after foliar application, reducing disease severity by 50%. The nanoparticles exhibited pH‐sensitive controlled release, specifically under the alkaline pH in plant phloem. The phloem loading of FLU@PGA was involved in an active transport mechanism at the organ level. The interaction of FLU@PGA with the plant amino acid transporter AtLHT1 (*A. thaliana* lysine histidine transporter) was observed to enhance transmembrane uptake at the cellular level.

The systemic plant immunomodulator β‐amino butyric acid has been shown to be ambimobile in tomato^26^ and lettuce.^27^ We are not aware of a fungicide that exudes from the root after foliar application.

Root exudation does occur with the nematicides oxamyl and fluensulfone. They are ambimobile, translocating both acropetally and basipetally, from foliage to root and root to foliage. Oxamyl was reported to exude from the roots of foliage‐treated plants.^28^

We conclude that OXPT is as an ambimobile fungicide. It can translocate in the plant acropetally from root to shoot and basipetally from shoot to root. OXPT can exude from the root to the soil. The exuded OXPT may be taken up by a neighboring plant thus performing a plant‐to‐plant mobility. OXPT is the first fungicide to show trans‐plant activity.

## AUTHOR CONTRIBUTIONS

Yigal Cohen undertook the biological experimentation and writing of the article. Michal Weitman performed the chemical analyses.

## CONFLICT OF INTEREST

The authors declare no conflict of interest.

## Data Availability

The data that support the findings of this study are available from the corresponding author upon reasonable request.
